# Altered States of Consciousness during an Extreme Ritual

**DOI:** 10.1371/journal.pone.0153126

**Published:** 2016-05-13

**Authors:** Ellen M. Lee, Kathryn R. Klement, James K. Ambler, Tonio Loewald, Evelyn M. Comber, Sarah A. Hanson, Bria Pruitt, Brad J. Sagarin

**Affiliations:** 1 Department of Psychology, Northern Illinois University, DeKalb, IL, United States of America; 2 University of Texas at Dallas, Richardson, TX, United States of America; 3 Department of Sociology, Georgia State University, Atlanta, GA, United States of America; University of Exeter, UNITED KINGDOM

## Abstract

Extreme rituals (body-piercing, fire-walking, etc.) are anecdotally associated with altered states of consciousness—subjective alterations of ordinary mental functioning (Ward, 1984)—but empirical evidence of altered states using both direct and indirect measures during extreme rituals in naturalistic settings is limited. Participants in the “Dance of Souls”, a 3.5-hour event during which participants received temporary piercings with hooks or weights attached to the piercings and danced to music provided by drummers, responded to measures of two altered states of consciousness. Participants also completed measures of positive and negative affect, salivary cortisol (a hormone associated with stress), self-reported stress, sexual arousal, and intimacy. Both pierced participants (pierced dancers) and non-pierced participants (piercers, piercing assistants, observers, drummers, and event leaders) showed evidence of altered states aligned with transient hypofrontality (Dietrich, 2003; measured with a Stroop test) and flow (Csikszentmihalyi, 1990; Csikszentmihalyi & Csikszentmihalyi, 1990; measured with the Flow State Scale). Both pierced and non-pierced participants also reported decreases in negative affect and psychological stress and increases in intimacy from before to after the ritual. Pierced and non-pierced participants showed different physiological reactions, however, with pierced participants showing increases in cortisol and non-pierced participants showing decreases from before to during the ritual. Overall, the ritual appeared to induce different physiological effects but similar psychological effects in focal ritual participants (i.e., pierced dancers) and in participants adopting other roles.

## Introduction

Extreme rituals (e.g., body-piercing, fire-walking) have been documented historically [1] and are widely practiced today [2]. Prior research has identified a number of effects of performing extreme rituals. Fire-walkers, for example, showed physiological synchrony with related observers [3], autobiographical memory deficits [4], and increases in happiness from before to after the ritual [2]. Likewise, performers of Kavadi, a high-ordeal ritual within the Hindu Thaipusam festival, showed increases in pro-social behavior after the ritual [5]. The experience of an altered state of consciousness is reported as another common occurrence during extreme rituals [6] and is often anecdotally documented [7, 8]. Altered states of consciousness have been described as “a qualitative alteration in the overall patterns of mental functioning so that the experiencer feels that his/her operations of consciousness are radically different from ordinary functioning” [9]. There are many different types of altered states, such as meditation, hypnosis, mental absorption, drug-induced states, spiritual possession, and religious ecstasy. Furthermore, the meaning attributed to an altered state is subjective and can depend on a variety of factors including context and personal characteristics [6, 10]. However, very few research studies have empirically demonstrated evidence of altered states using direct and indirect measures during extreme rituals in naturalistic settings. In one study, 44 individuals who took part in the Thaipusam Kavadi ritual retrospectively reported on their experience of dissociative symptoms during the ritual, including amnesia, absorption, depersonalization, and derealisation [10]. The results demonstrated that all but one participant evidenced dissociative symptoms during the ritual and that these experiences were not typical of the participants’ everyday lives. Further, individuals who experienced a high number of dissociative symptoms during the ritual also reported lower pain intensity during the piercing.

Other studies have attempted to mimic ritual like conditions in laboratory settings to study participants’ cognitive and affective responding. In one study, for example, researchers investigated via fMRI how the brain patterns of highly hyponotically susceptible participants would change in response to varying levels of a loss of self-control in order to replicate the experiences of dissociation and possession; i.e., how much they thought an external force was controlling their hand movements and how much they lost their sense of self [11]. Among the many results, it was demonstrated that in the conditions where participants were told their involuntary hand movements were controlled by an alien force, compared to the condition where the movements were caused by a malfunctioning machine, there was an “increased connectivity between primary motor cortex (M1) and brain regions involved in attribution of mental states and representing the self in relation to others” (p. 107). The authors suggest that many brain systems are likely involved during experiences of a loss of self-control. Another fMRI study looked at the effects of performing formalized prayer speech (frequently rehearsed and rigidly performed; e.g., Lord’s prayer) and improvised prayer speech (personal prayer) among orthodox Dutch Christians who believe in a personal, real god capable of responding to requests [12]. The findings demonstrated that personal praying and formalized praying activated different neural regions. For example, the personal prayer condition activated areas strongly associated with social cognition and “theory of mind” (the medial prefrontal cortex and the temporo-parietal junction), whereas the formalized prayer condition activated a number of areas in the opposite contrast (the dorsolateral prefrontal cortex and the cerebellum). Taken together, these studies suggest that brain activity is likely to change under circumstances where individuals are experiencing ritual- like conditions but the various conditions potentially change an individual’s subjective experience.

The goals of this study were to test whether participants engaged in an extreme ritual in a naturalistic setting would evidence signs of altered states of consciousness, to examine other physiological and affective effects of the ritual, and to determine whether these effects varied based on the role the individual performed within the ritual. To this end, we took a multi-method approach to this study, including utilizing various psychological self-report measures, a measure of cognitive functioning, and a measure of physiological stress.

Data collection took place at the “Dance of Souls”, a ritual conducted on the last day of the annual Southwest Leather Conference in Phoenix, Arizona in which participants receive temporary piercings with hooks or weights attached to the piercings and dance to music provided by drummers. The Dance of Souls is reported to have been inspired by the Plains Native American Sundance (a.k.a. O-Kee-Pa ceremony) [1] and the Hindu Thaipusam festival of the Tamil communities [7]. In both these traditions participants pierce their flesh, using body stress to induce ecstatic states. The introduction of hook pulls/ball dances to the sadomasochistic and body modification communities is often attributed to Fakir Musafar [13], with the Dance of Souls tracing its roots to ball dances led by Musafar and Cleo Dubios in 1999 and 2000. The Dance of Souls itself became an annual event in 2006, led first by Elwood Reid, a protégé of Musafar, and subsequently by Lauren Ide, a protégé of Reid who also studied with Musafar.

The study of the Dance of Souls enabled us to extend prior research on extreme rituals in a number of ways. First, prior research on extreme rituals has typically measured responses of focal ritual participants (e.g., fire-walkers) and, in some studies, responses of observers. At the Dance of Souls, a number of individuals adopting other roles (e.g., piercers, drummers, dance leaders, and observers) also volunteered for the study, enabling a comparison of the effects of the ritual on focal ritual participants (i.e., pierced dancers) with the effects of the ritual on non-pierced participants in other roles. Second, pierced dancers in the Dance of Souls typically pull against their piercings for two to three hours, providing a useful comparison with shorter duration rituals such as fire-walking. Third, the length of the ritual and the demographics of ritual participants enabled us to use a longer and more complete set of validated instruments to assess the variables of interest (e.g., the Positive and Negative Affect Scale [14], the Inclusion of Other in Self Scale [15], a 40-trial Stroop test, etc.). Last, in contrast to prior studies of explicitly religious rituals [5] or annual community rituals [2,3], the Dance of Souls took place on the final day of a four day leather and Bondage-Discipline/Dominance-Submission/Sadism-masochism (BDSM) conference, thus providing a unique setting in which to investigate the meanings participants attributed to the ritual event.

We predicted that an extreme ritual would induce two distinct, role-specific altered states of consciousness. First, we predicted that pierced participants would enter an altered state aligned with transient hypofrontality [16]. The theory of transient hypofrontality rests on two premises: (a) the brain has limited resources, and brain structures, systems, and areas compete for these resources; and (b) the subjective experience of consciousness is a process. When activities, such as exercise, increase the demands on brain areas responsible for basic sensory and perceptual processes, autonomic nervous system regulation, and motor output, the brain does not receive additional blood flow. Rather, the brain down-regulates certain regions to increase blood flow to currently important areas, and data suggest that the frontal cortex and the prefrontal cortex are consistently down-regulated in this way [17, 18]. The dorsolateral prefrontal cortex is heavily responsible for working memory and sustained attention, thus down regulation to this area can lead to changes in subjective perception of reality, including time distortions, disinhibition from social constraints, and changes in focused attention. Other theorized subjective experiences of transient hypofrontality include reductions in pain, living in the here and now, little active decision making, little active logic, and feelings of floating and peacefulness [16]. This change in subjective perception via down-regulation is often interpreted positively, for example: “exercise may provide relief from stress, anxiety, and negative thinking patterns by running on ‘safe mode’ the very thinking structure that instigates these mental troubles- the prefrontal cortex” [19], p. 522–523. The theory provides a mechanistic explanation for why engaging in activities within the moderate, aerobic range can contribute positively to mental health. Transient hypofrontality is hypothesized to be the underlying mechanism in many documented altered states, such as runner’s high, meditation, hypnosis, and daydreaming. The subjective experience, and thus characterization, of the altered states depends on the severity of the prefrontal hypofunction. Therefore, one measurable implication of this theory is that an individual experiencing an altered state would evidence impaired prefrontal cognitive functioning. Given the consistency between transient hypofrontality and the descriptions of individuals involved in extreme rituals [6, 7, 8, 10], we posited that transient hypofrontality would be relevant to the Dance of Souls. Indeed, the earlier discussed dissociative experiences of the Thaipusam participants are consistent with the experiences associated with transient hypofrontality [10]: absorption, depersonalization, derealization, and changes in time perception.

Although an fMRI would be a direct test of transient hypofrontality by measuring blood flow to the dorsolateral prefrontal cortex, many activities, such as the Dance of Souls, involve movement that precludes the use of fMRI, and thus transient hypofrontality is typically indirectly examined with prefrontal-dependent cognitive tests [19]. In one study employing the use of a prefrontal-dependent cognitive test, the Wisconsin Card Sorting Task, and a non prefrontal-dependent measure, the Brief Kaufman Intelligence Test, Dietrich and Sparling [19] discovered that athletes engaging in moderate exercise (running or cycling for 20 minutes before testing) showed impaired prefrontal-cognition, but not impaired general intelligence, compared to sedentary controls. As noted by Dietrich and Sparling, the Wisconsin Card Sorting Task has strong training effects, so in their second study they used a different cognitive test for a repeated-measures design, the Paced Auditory Serial Addition Task. As in the first study, they also used another measure of general intelligence, the Peabody Picture Vocabulary Test. The results from the second study demonstrated that athletes engaging in moderate exercise (running for 40 minutes before testing) showed impaired prefrontal-cognition, but not impaired general intelligence, compared to when they were not engaged in such exercise. Another study investigated transient hypofrontality not only before and during exercise but also afterwards to examine the effects various exertion levels have on executive functioning [17]. In a repeated measures design, participants were instructed to exercise on a stationary bicycle at 75% of their ventilatory threshold (VT) or at their VT and complete prefrontal-dependent cognitive tasks. The results showed that participants evidenced impaired executive functioning at both levels during exercise, but impairment was only seen immediately after exercise for the VT condition only. Given the physical pain of the piercing(s) and the physically demanding activity of pulling against the piercings, we expected that pierced dancers would show greater cognitive functioning impairment during the ritual compared to those not being pierced. Because we employed a repeated-measures design, we also used a prefrontal-dependent cognitive test that does not have strong training effects: the Stroop test. Additionally, unlike the above mentioned cognitive tests [19], the Stroop can be administered quickly (1–2 minutes vs. 25 minutes). We developed a Stroop application for use on tablets, thus making the test highly portable and easy to administer. Prior research has shown that the Stroop is sensitive to altered states of consciousness such as hypnosis [20] and has been used as a measure of executive functioning in other exercise studies [21, 22]. Furthermore, although the Stroop task is an indirect measure of an altered state of consciousness, it is an objective measure of changes in mental processing and thus not as susceptible to being faked compared to subjective measures like self-reports.

Second, we predicted that individuals conducting and facilitating the ritual would enter an altered state aligned with flow [23,24]. Flow is typically described as a positive mental state in which someone experiences an intense absorption and focus during an activity in which they have the appropriate skills to meet the challenges of that activity. Flow has been documented with musicians [25], athletes [26, 27], and within educational settings [28]. Flow is composed of multiple dimensions, some related to optimal performance (e.g., clear goals, sense of control), others related to autotelic absorption (e.g., loss of self-consciousness, transformation of time) [29]. We expected that non-pierced participants, particularly those conducting service-oriented activities during the ritual (e.g., piercers, drummers, and ritual leaders), would retrospectively self-report higher levels of flow during the Dance than would pierced dancers in dimensions associated with optimal performance but that all participants, pierced and non-pierced, would show similar levels of flow in dimensions associated with autotelic absorption. We make these predictions based on the high levels of skill and focus required to successfully perform activities such as piercing and drumming. Thus, the activities engaged in by many non-pierced participants are the types of high-skill/high-challenge activities theorized to facilitate flow [23, 24] and embodied in the optimal performance dimensions of flow. In contrast, we predicted that the autotelic absorption dimensions of flow (encompassing enjoyment, loss of self-consciousness, transformation of time, and action-awareness merging) would characterize the experience of both pierced participants and non-pierced participants, as all types of participants are expected to find the Dance of Souls to be absorbing and enjoyable. Furthermore, the absorption aspect of flow is similar to what would be predicted by transient hypofrontality in regards to the subjective effects of the Dance for all participants, whereas the performance aspect (in particular, being in control and having clear goals) seemed less relevant to pierced participants. Flow has often been measured retrospectively via self-report using instruments such as the Flow State Scale [29, 30]. Therefore, it was of interest to have both a direct (but subjective) measure and an indirect (but objective) measure of altered states of consciousness to investigate the effects of the ritual.

We also predicted that pierced and non-pierced participants would show a range of affective and physiological responses to the ritual. Specifically, based on Fischer et al.’s [2] finding that fire-walkers’ happiness increased from before to after the fire-walking, we expected an increase in positive affect, a decrease in negative affect, and a decrease in self-reported stress from before to after the event. Indeed, theorists propose that pain-related rituals can paradoxically help individuals cope with life stressors or to achieve some personal goal like cleansing [10, 31]. However, the way an individual emotionally responds to a ritual has been shown to be different depending on the role they assume [2, 32]. For example, in the Fischer et al. fire-walking study [2], it was shown that participants who watched their relatives engage in the ritual reported feeling more fatigue post-ritual compared to fire-walkers and non-related spectators, whereas the fire-walkers reported the greatest happiness post ritual compared to related and non-related spectators. In another study, independent raters viewed a series of images from a videotaped fire-walking ritual depicting a walker/passenger dyad (a fire-walker carrying a passenger throughout ritual) in five phases of the fire walk: entry, early fire walk, middle fire walk, late fire walk, and completion [32]. The results demonstrated that arousal increased over time for both fire-walkers and passengers but most intensely for fire-walkers, and furthermore the valence was different; fire-walkers exhibited an increase in negative arousal on average whereas passengers exhibited an increase in positive arousal. Therefore, we were interested in examining the effects of the ritual separately for the various roles individuals took on during the Dance, and we predicted the affective changes post ritual would be strongest among the pierced participants.

We additionally predicted that feelings of intimacy would increase for all Dance participants for two reasons: a) in his explication of transient hypofrontality, Dietrich discusses how individuals experiencing an altered state (like meditation or hypnosis) may report changes in their sense of self [16] and thus it seemed plausible that people would report a greater connection with others, and b) rituals have been theorized to promote social bonds [8, 31]. Furthermore, others have posited that collective rituals increase social cohesion through group identification and a blurring of the boundary between self and other [33].

In contrast to these affective responses, we predicted that pierced and non-pierced individuals would differ in their physiological responses to the ritual. In particular, we expected that pierced individuals would show increases in cortisol, a hormone associated with physiological stress, from before to during the event, given the pain of the piercings, but that non-pierced individuals would show no changes in cortisol. Cortisol has been shown to rise in response to both negative and positive experiences [34]. Further, we were interested in comparing participants’ physiological stress response to their psychological responses. Particularly, and consistent with our prior hypothesizing, we predicted that there would be a misalignment between physiological stress and self-reported psychological stress for pierced participants, in that physiological stress would increase and psychological stress would decrease.

Finally, we were interested in investigating participants’ conceptualization of the Dance of Souls. We expected some degree of sadomasochistic conceptualization of the ritual because (a) the event occurred within the context of a BDSM conference, and (b) aspects of the activities align with aspects of sadomasochistic practice (i.e., the enjoyment of intentional and consensual infliction and receipt of pain). We expected some degree of sexual conceptualization of the ritual because (a) sexuality is a strong component of BDSM behavior [35] (although sexual activity does not always occur during BDSM activities) [36], and (b) the event involves partial or full nudity by some participants. Lastly, we expected some degree of spiritual conceptualization of the ritual because (a) the event was inspired by other extreme rituals in religious contexts, and (b) BDSM community literature indicates that some individuals may use sadomasochistic practices for spiritual purposes [37, 38]. Thus, we sought to investigate and compare these three conceptualizations: how sadomasochistic, sexual, and spiritual participants interpreted the Dance of Souls.

### Ethics Statement

This study was reviewed and approved by Northern Illinois University’s Institutional Review Board (Protocol # HS11-0410). A written record of informed consent was waived due to confidentiality concerns and instead a verbal consent procedure was approved. Thus, oral informed consent was obtained from the participants by the researchers. This verbal consent was recorded on the master participant list.

## Results

A total of 83 participants began the study, consisting of 47 pierced dancers, seven piercers, six piercing assistants, seven observers, three drummers, seven event/spiritual leaders, and six unknown/missing roles (e.g.,“spiritual growth journey”, or missing pre and/or post surveys). Given the small sample sizes in some of the roles, we created one group of pierced dancers and another group of non-pierced participants. This resulted in 47 pierced participants and 36 non-pierced participants, and subsequent analyses were conducted using this role dichotomization. The six participants with unknown/missing roles were categorized as non-pierced participants. Alternate analyses that exclude these participants did not substantively change the results because many of them did not provide a full set of measures; however, we opted to include these participants in the final sample in order to retain as much power as possible.

### Stroop

**Fig 1:**
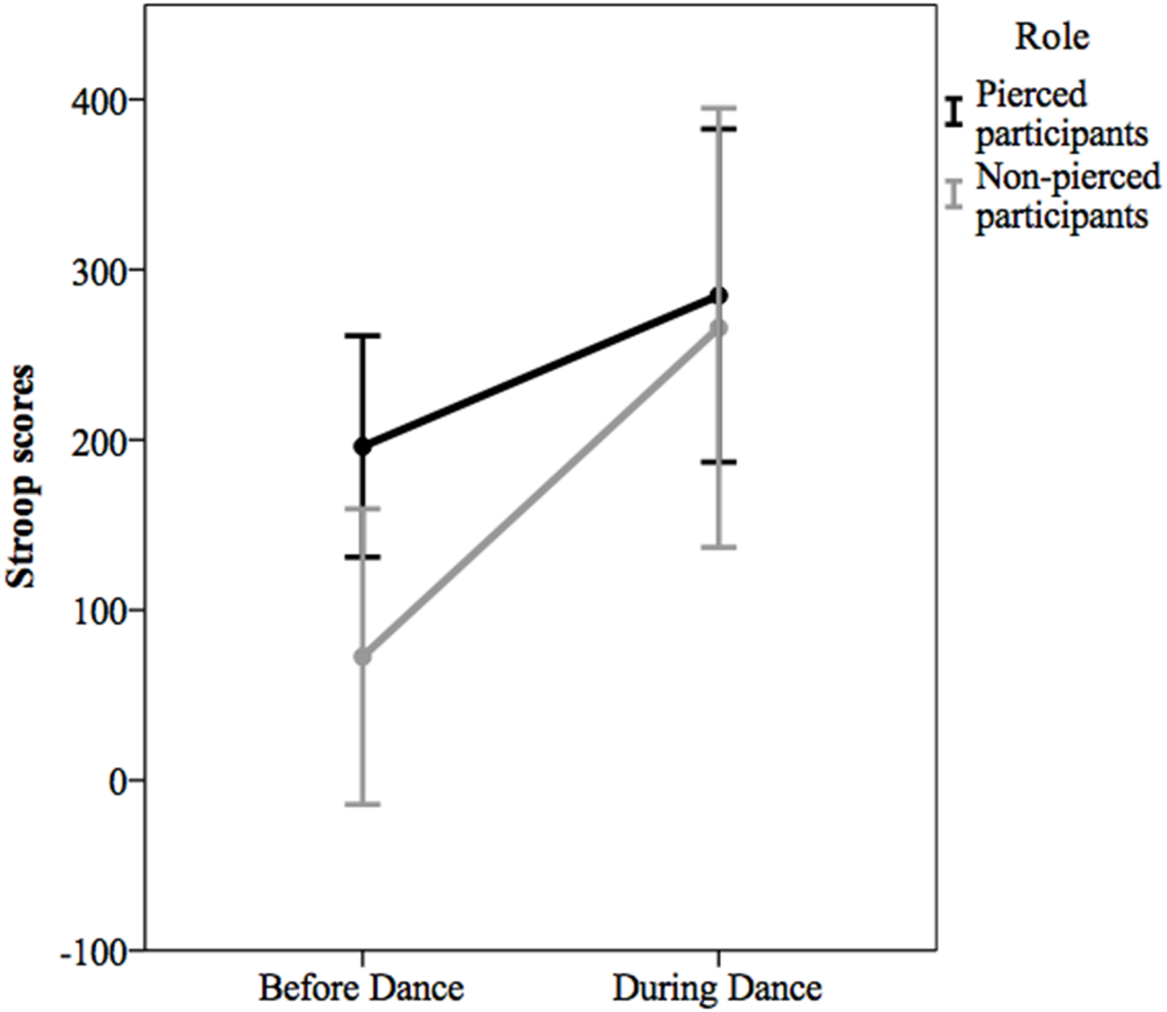
Stroop scores by dichotomized role. Stroop scores reflect the difference between the mean latencies on incongruent trials (*red* in green type) and the mean latencies on congruent trials (*red* in red type). Included in the analyses were the 35 pierced participants and 24 non-pierced participants who completed both baseline and during-Dance Stroop scores. Error bars are 95% confidence intervals.

A repeated-measures ANOVA revealed that Stroop latency scores increased significantly from baseline (*M* = 134.37, *SE* = 25.97) to during the event (*M* = 275.30, *SE* = 38.87), *F*(1, 57) = 8.42, *p* = .005, partial η^2^ = .13, 95% CI [43.65, 238.19], indicating impaired executive functioning. The main effect of role was non-significant, *F*(1, 57) = 2.52, *p* = .12, partial η^2^ = .04, and there was not a significant role by time interaction, *F*(1, 57) = 1.16, *p* = .29, partial η^2^ = .02 (Fig 1). Contrary to predictions, pierced and non-pierced participants did not differ significantly in their degree of impaired executive functioning from baseline to during the Dance. We had predicted that pierced participants would evidence a greater change in Stroop performance compared to non-pierced participants, but the time by role interaction was not significant. We also examined the baseline Stroop scores and determined that the participants who did not get pierced (*M* = 72.63, *SD* = 205.59) had marginally significantly lower baseline Stroop scores than did individuals who would eventually get pierced (*M* = 169.12, *SD* = 189.27), *t* (57) = 1.85, *p* = .07, 95% CI [-200.52, 7.53].

### Flow

**Fig 2:**
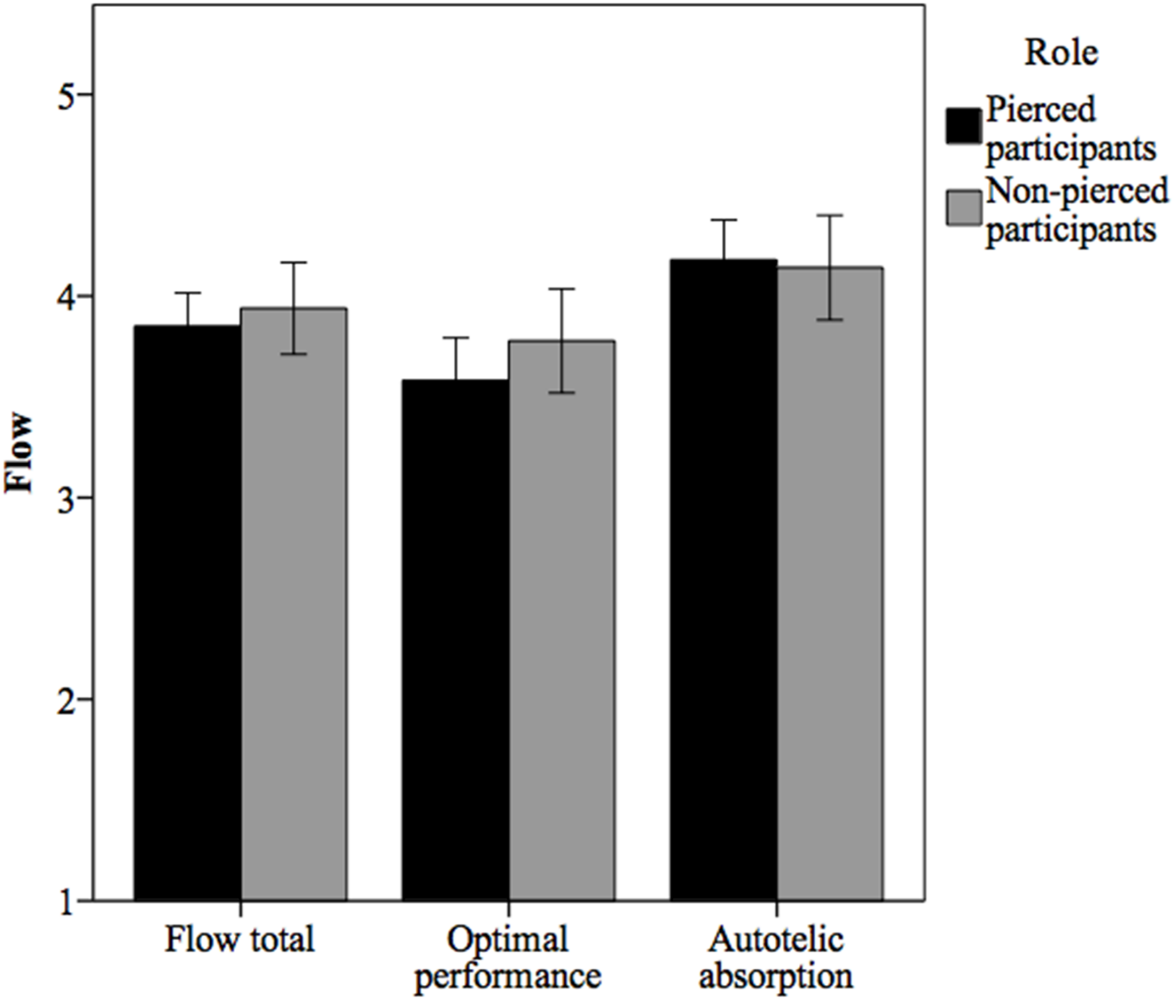
Flow scores by dichotomized role. The optimal performance factor consists of Challenge-Skill Balance, Clear Goals, Unambiguous Feedback, Concentration on Task at Hand, and Sense of Control. The autotelic absorption factor consists of Action-Awareness Merging, Loss of Self-Consciousness, Transformation of Time, and Autotelic Experience. Error bars are 95% confidence intervals.

Items on the 36-item Flow State Scale were answered on a 1 (*strongly disagree*) to 5 (*strongly agree*) scale with scores higher than 3 (*neither agree nor disagree*) indicating flow [30]. As the means in Fig 2 illustrate, both pierced and non-pierced participants retrospectively reported post-Dance that they had experienced high levels of total flow during the Dance; flow total for both pierced and non-pierced participants (*M* = 3.89, *SD* = .55) was significantly higher than 3, *t*(68) = 13.31, *p* <.001, 95% CI [.75, 1.02]. Contrary to our predictions, however, a *t*-test demonstrated that total flow did not differ significantly between pierced participants and non-pierced participants, *t*(67) = 0.66, *p* = .51, 95% CI [-1.80, .36] (Fig 2). To further test our hypotheses that the dimensions of flow related to optimal performance would be more relevant to non-pierced participants compared to pierced participants, but enjoyment would be relevant to all participants, we ran an exploratory factor analysis using principle components analysis on the nine dimensions of flow. The Scree Plot criterion suggested a two-factor solution. Using Direct Oblimin rotation (a non-orthogonal rotation method), the two factors that emerged were (a) Challenge-Skill Balance, Clear Goals, Unambiguous Feedback, Concentration on Task at Hand, and Sense of Control, and (b) Action-Awareness Merging, Loss of Self-Consciousness, Transformation of Time, and Autotelic Experience. We labeled the first factor *optimal performance* and the second factor *autotelic absorption*. Contrary to our predictions, pierced participants and non-pierced participants did not differ significantly on either optimal performance, *t*(67) = 1.21, *p* = .23, 95% CI [-1.28, .52], or autotelic absorption, *t*(67) = -0.24, *p* = .81, 95% CI [-.35, .28].

### Cortisol

**Fig 3:**
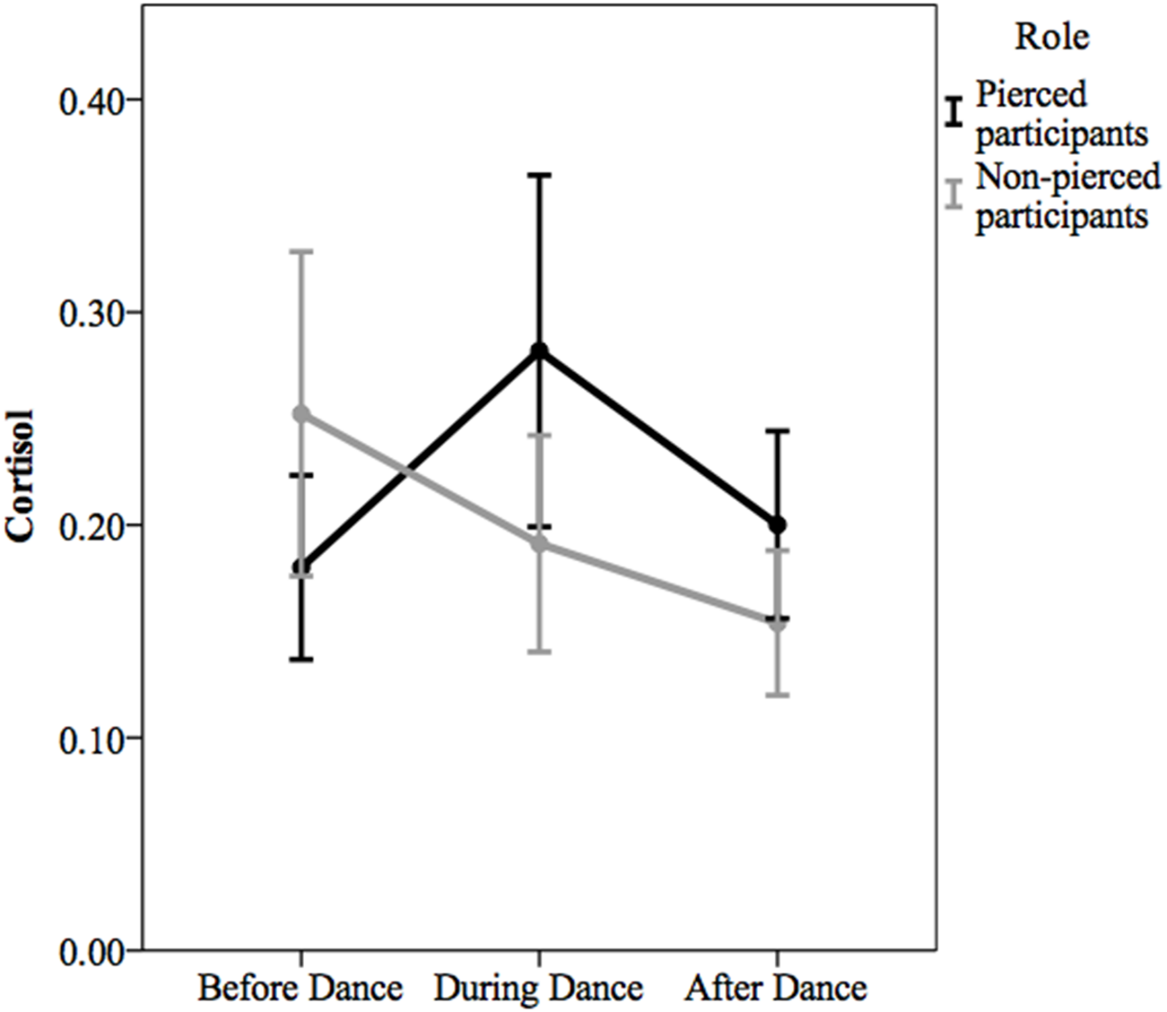
Cortisol levels by dichotomized role. A total of 18 pierced and 25 non-pierced participants provided saliva samples at all three time points. Error bars are 95% confidence intervals.

For cortisol, a repeated- measures ANOVA revealed a marginally significant main effect of time, *F*(2, 82) = 2.98, *p* = .06, partial η^2^ = .07 (Fig 3). The main effect of role was non-significant, *F*(1, 41) = .54, *p* = .47, partial η^2^ = .01. The interaction between role and time was significant, *F*(2, 82) = 5.78, *p* = .004, partial η^2^ = .12. Mauchly’s test of Sphericity indicated that the assumption of sphericity had not been violated, χ^2^ (2) = 2.83, *p* = .24. Consistent with our hypothesis, the pattern of cortisol across time was different for pierced vs. non-pierced participants. Pierced participants exhibited an increase in cortisol from pre-Dance to during-Dance and a decrease in cortisol from during-Dance to post-Dance. In contrast, non-pierced participants’ cortisol level was highest before the Dance began, declining from pre-Dance to during-Dance and from during-Dance to post-Dance.

### Self-report measures and Dance interpretations

A series of repeated-measures ANOVAs were conducted using the measures of positive and negative affect, psychological stress, psychological sexual arousal, and the IOS (Table 1). Contrary to our predictions, positive affect did not change significantly from before the Dance to after the Dance, *F*(1, 65) = 1.66, *p* = .20, partial η^2^ = .02. The main effect of role was non-significant, *F*(1, 65) = 2.38, *p* = .13, partial η^2^ = .03, and the role by time interaction was also non-significant, *F*(1, 65) = 1.54, *p* = .22, partial η^2^ = .02.

**Table 1:** Self-report Measures and Dance Interpretations in Pierced and Non-pierced Participants.

Measure	Role	Pre-Dance		Post-Dance	
		*Mean* (*SD*)	95% CI	*Mean* (*SD*)	95% CI
Positive affect	Pierced	3.54 (.76)	[3.30–3.78]	3.54 (.79)	[3.27–3.80]
	Non- pierced	3.92 (.72)	[3.64–4.20]	3.68 (.89)	[3.37–4.00]
Negative affect	Pierced	1.69 (.54)	[1.52–1.87]	1.20 (.31)	[1.11–1.30]
	Non- pierced	1.45 (.57)	[1.25–1.66]	1.19 (.29)	[1.08–1.30]
Stress	Pierced	1.95 (1.00)	[1.63–2.27]	1.18 (.45)	[1.01–1.35]
	Non- pierced	1.71 (1.01)	[1.34–2.09]	1.32 (.61)	[1.12–1.52]
Sexual arousal	Pierced	1.72 (.86)	[1.44–1.99]	2.49 (1.36)	[2.05–2.93]
	Non- pierced	1.71 (.85)	[1.39–2.04]	2.36 (1.42)	[1.84–2.88]
IOS	Pierced	3.77 (1.65)	[3.21–4.33]	4.28 (1.82)	[3.73–4.84]
	Non- pierced	4.36 (1.87)	[3.70–5.02]	4.93 (1.63)	[4.27–5.59]
How spiritual	Pierced	3.85 (1.30)	[3.47–4.23]	3.85 (1.18)	[3.48–4.22]
	Non- pierced	3.92 (1.08)	[3.55–4.29]	3.97 (1.21)	[3.53–4.41]
How sadomasochistic	Pierced	2.99 (1.26)	[2.63–3.35]	2.68 (1.16)	[2.32–3.04]
	Non- pierced	2.14 (1.10)	[1.76–2.52]	2.19 (1.06)	[1.80–2.58]
How sexual	Pierced	2.08 (1.05)	[1.78–2.38]	2.49 (1.26)	[2.10–2.88]
	Non- pierced	2.30 (1.18)	[1.90–2.70]	2.38 (1.29)	[1.91–2.85]

*Note*. A total of 39 pierced individuals and 28 non-pierced individuals completed the PANAS, stress, sexual arousal, and IOS measures. A total of 40 pierced individuals (39 on how spiritual) and 29 non-pierced individuals answered the pre-Dance interpretation questions, whereas 46 pierced individuals and 33 non-pierced individuals answered the post-Dance interpretation questions. *SD* = standard deviation. 95% CI = 95% confidence intervals.

As predicted, negative affect significantly decreased from pre-Dance (*M* = 1.57, *SE* = .07) to post-Dance (*M* = 1.20, *SE* = .03), *F*(1, 65) = 41.45, *p* < .001, partial η^2^ = .38, 95% CI [.26, .50]. The main effect of role was not significant, *F*(1, 65) = 1.87, *p* = .18, partial η^2^ = .03, but there was a marginally significant role by time interaction, *F*(1, 65) = 3.77, *p* = .06, partial η^2^ = .05. Consistent with our predictions, all participants reported a decrease in negative affect from before the Dance to after the Dance, and this change was greatest in pierced participants.

Psychological stress also significantly decreased pre-Dance (*M* = 1.83, *SE* = .12) to post-Dance (*M* = 1.25, *SE* = .07), *F*(1, 65) = 24.00, *p* < .001, partial η^2^ = .27, 95% CI [.34, .82], consistent with our hypothesizing. The main effect of role not significant, *F*(1, 65) = .08, *p* = .77, partial η^2^ = .001, nor was there was a significant role by time interaction, *F*(1, 65) = 2.52, *p* = .12, partial η^2^ = .03. It appeared that all participants report a reduction in stress from before to after the Dance, despite our hypothesis that this effect would be greatest among pierced participants.

Consistent with our predictions, self-other overlap significantly increased pre-Dance (*M* = 4.06, *SE* = .22) to post-Dance (*M* = 4.61, *SE* = .22), *F*(1, 65) = 5.66, *p* = .02, partial η^2^ = .08, 95% CI [.08, .99]. There was a non-significant main effect of role, *F*(1, 65) = 2.84, *p* = .10, partial η^2^ = .04, and the role by time interaction was non-significant, *F*(1, 65) = .02, *p* = .90, partial η^2^ = .00. The pattern of increased intimacy from before the Dance to after the Dance was similar for both pierced and non-pierced participants.

Psychological sexual arousal significantly increased pre-Dance (*M* = 1.72, *SE* = .11) to post-Dance (*M* = 2.42, *SE* = .17), *F*(1, 65) = 20.05, *p* < .001, partial η^2^ = .24, 95% CI [.39, 1.02]. The main effect of role was not significant, *F*(1, 65) = .08, *p* = .78, partial η^2^ = .001, nor was the role by time interaction significant, *F*(1, 65) = .16, *p* = .69, partial η^2^ = .002. All Dance participants reported an increase in psychological sexual arousal from before to after the Dance.

Before the Dance began, participants were asked how spiritual, sadomasochistic, and sexual they anticipated the Dance would be for them, and they were asked these same questions in past tense after the Dance was over. Mauchly’s test of Sphericity indicated that the assumption of sphericity had not been violated, χ^2^ (2) = 3.75, *p* = .15. An examination of the means revealed that participants anticipated that the Dance would be more spiritual (*M* = 3.88, *SD* = 1.20, 95% CI [3.61, 4.45]) than sadomasochistic (*M* = 2.63, *SD* = 1.26, 95% CI [2.35, 2.91]) and more sadomasochistic than sexual (*M* = 2.17, *SD* = 1.11, 95% CI [1.92, 2.41]), *F*(2, 156) = 52.80, *p* < .001. Somewhat similar results were found with the post-Dance interpretations. Mauchly’s test of Sphericity indicated that the assumption of sphericity had not been violated, χ^2^ (2) = 1.88, *p* = .39. Participants viewed the Dance as more spiritual (*M* = 3.90, *SD* = 1.19, 95% CI [3.61, 4.18]) than sadomasochistic (*M* = 2.49, *SD* = 1.13, 95% CI [2.21, 2.77]) or sexual (*M* = 2.45, *SD* = 1.27, 95% CI [2.14, 2.75]), *F*(2, 134) = 38.33, *p* < .001.

We also tested whether these interpretations differed by role. There were no differences between pierced and non-pierced participants on the anticipated spirituality or sexuality of the Dance, *t*(77) = .28, *p* = .78, 95% CI [-.47, .62], *t*(77) = .90, *p* = .37, 95% CI [-.28, .73] respectively. People who were planning to get pierced, however, did anticipate the Dance to be more sadomasochistic than did people not planning to get pierced, *t*(77) = 3.13, *p* = .002, 95% CI [1.39, .31]. Similar results were found after the Dance. There were no significant differences on post-Dance spirituality or sexuality between pierced and non-pierced individuals, *t*(66) = .41, *p* = .68, 95% CI [-.46, .70], *t*(67) = .35, *p* = .73, 95% CI [-.73, .51], respectively. There was a marginally significant difference on post-Dance sadomasochism between pierced individuals and non-pierced individuals, however, with pierced participants perceiving the Dance to have been more sadomasochistic than did non-pierced participants, *t*(67) = 1.78, *p* = .08, 95% CI [-1.03, .06].

### Exploratory Investigation of the Data

**Fig 4:**
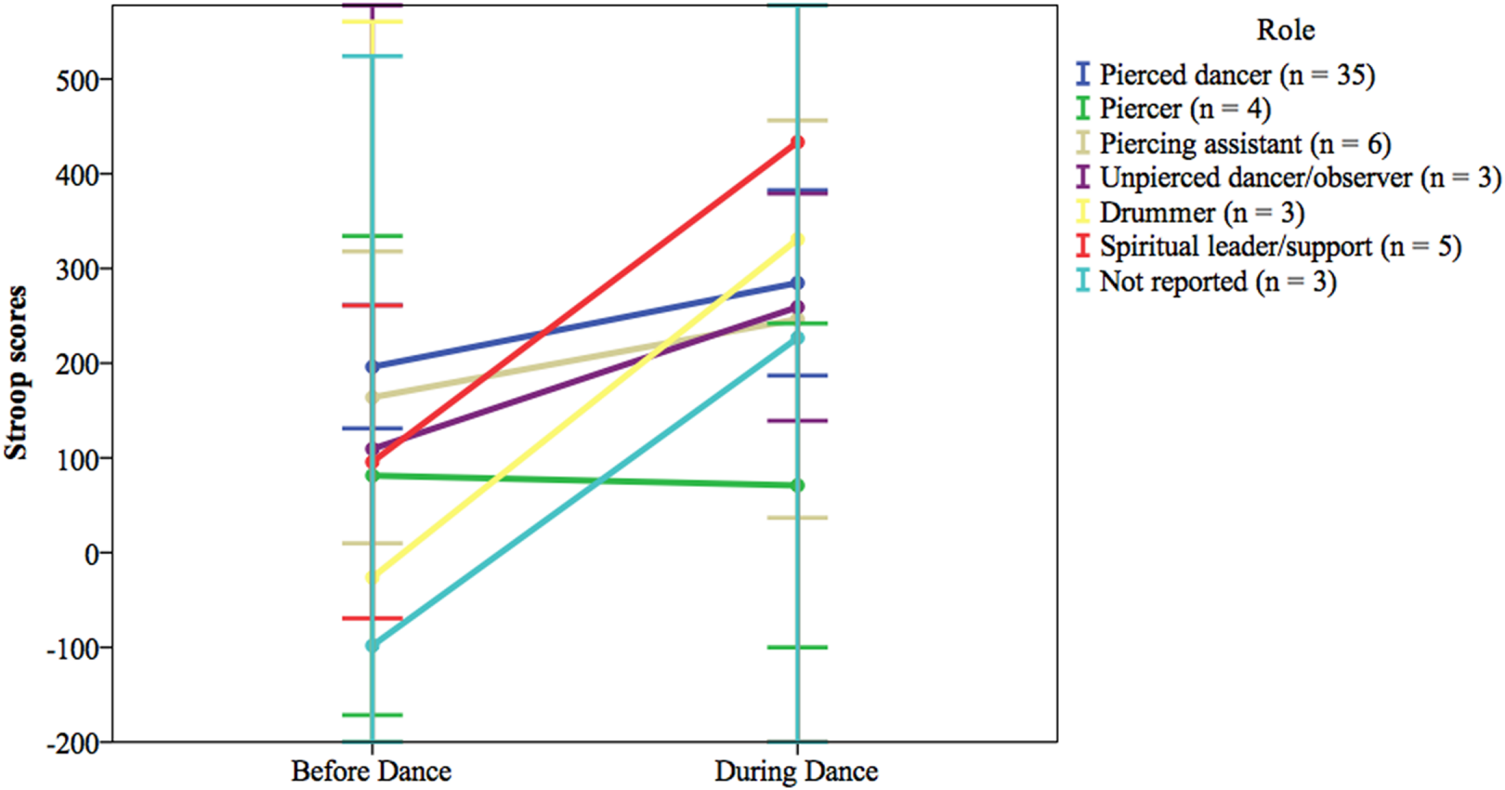
Stroop scores by all roles. The following participants completed both baseline and during-Dance Stroop task: 35 pierced dancers, 4 piercers, 6 piercing assistants, 3 non-pierced dancers/observers, 3 drummers, 5 spiritual leaders, 3 unknown/missing role. Error bars are 95% confidence intervals. The following upper error bars were truncated at 600: Before Dance/Unpierced dancer/observer, During Dance/Drummer, During Dance/Spiritual leader/support, and During Dance/Not reported. The following lower error bars were truncated at -200: Before Dance/Unpierced dancer/observer, Before Dance/Drummer, Before Dance/Not reported, During Dance/Drummer, During Dance/Spiritual leader/support, and During Dance/Not reported.

**Fig 5:**
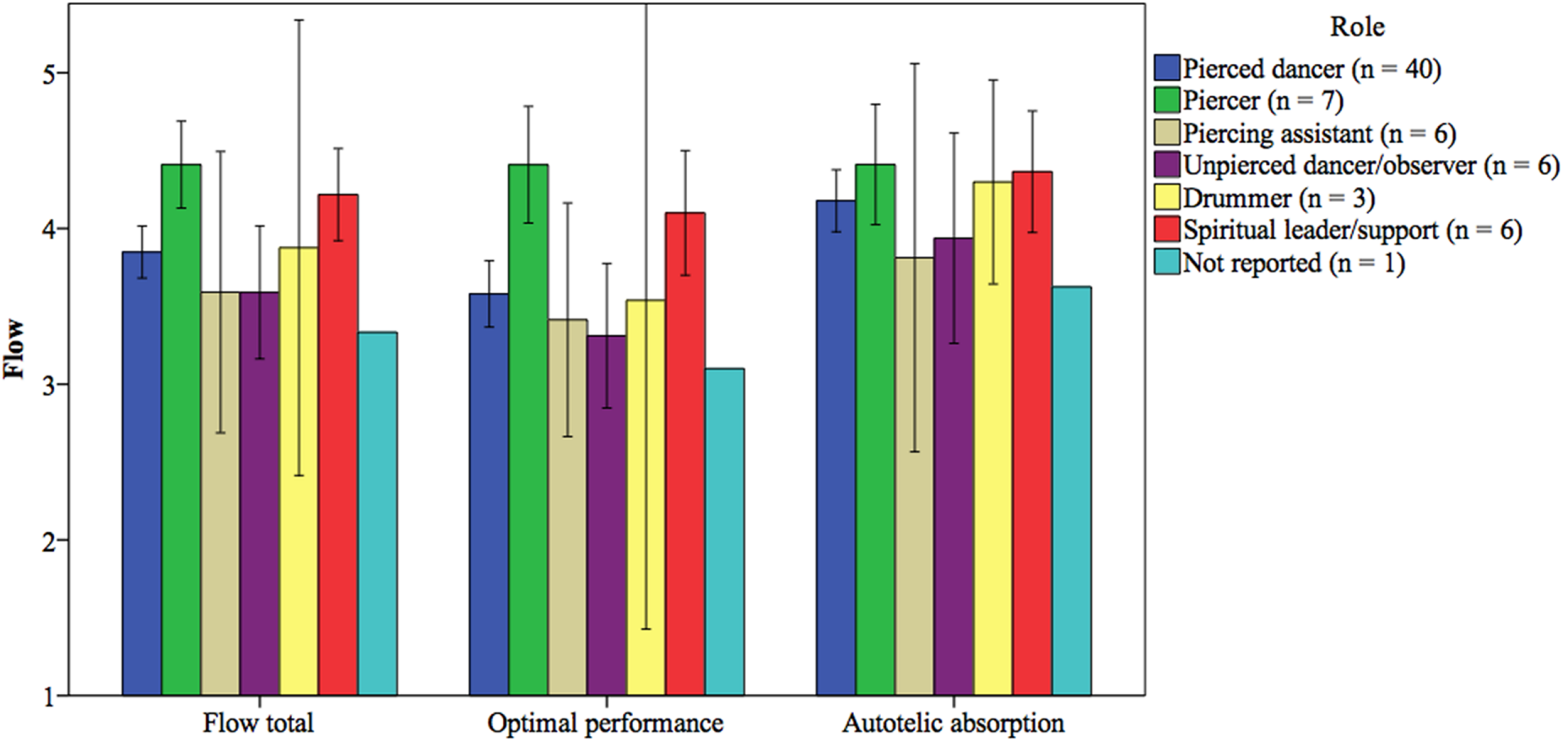
Flow scores by all roles. The following participants completed the retrospective self-report flow on the post-Dance survey: 40 pierced dancers, 7 piercers, 6 piercing assistants, 6 non-pierced dancers/observers, 3 drummers, 6 spiritual leaders, 1 unknown/missing role. Error bars are 95% confidence intervals.

Unfortunately, the small sample sizes of the roles within the non-pierced group precluded our ability to run separate analyses for these participants. However, because of our interest in examining specific group differences, and because prior literature suggests that such differences will occur, we present the means for the Stroop task and flow measure separated by role within the non-pierced participant group (Figs 4 and 5). These means should be interpreted with caution, of course, but we hope they will be useful to researchers in informing future hypotheses and in highlighting the need for increased sample size within each role.

An examination of the means within the non-pierced participant group revealed that all roles but one showed an increase in Stroop scores from baseline to during the event (Fig 4). The one exception were the four piercers whose Stroop performance actually improved from baseline (*M* = 81.35, *SD* = 158.92) to during-Dance (*M* = 70.94, *SD* = 107.48).

As with the Stroop scores, we examined the flow means within the non-pierced participant group (Fig 5). Across roles, the seven piercers had the highest total flow (*M* = 4.41, *SD* = 0.30), as well as the highest optimal performance (*M* = 4.41, *SD* = 0.41) and autotelic absorption (*M* = 4.41, *SD* = 0.42).

We also explored gender and religious orientation as potential additional predictors in the original analyses. Because we collected religion in an open-ended format, we first coded these data into religious categories (see Method section). Subsequently, we created a dichotomized grouping variable for those with a religious orientation (who indicated believing in some religion or being “spiritual”) versus those without a religious orientation (who indicated atheism, agnostic, none, or the answers were missing or not clear).

For all dependent variables (Stroop, flow, cortisol, positive affect, negative affect, psychological stress, sexual arousal, self-other overlap, and Dance interpretation), we ran alternative analyses that included gender or religious orientation along with the other independent variables (time and role). There were no significant main effects of gender for Stroop, flow, cortisol, positive affect, negative affect, psychological stress, sexual arousal, self-other overlap, or Dance interpretation. There were no significant main effects of religious orientation for Stroop, flow, cortisol, positive affect, negative affect, psychological stress, sexual arousal, or self-other overlap.

With respect to the effects of religious orientation on Dance interpretation, there were no significant differences between religious participants and non-religious participants on how sadomasochistic or sexual they anticipated the Dance to be. However, religious participants anticipated the Dance to be more spiritual (*M* = 4.20, *SD* = .98) than did non-religious participants (*M* = 3.18, *SD* = 1.36), *F*(1, 77) = 14.49, *p* < .001. A similar pattern was found with the post-Dance interpretation questions; there were no significant differences between religious participants and non-religious participants on how sadomasochistic or sexual they found the Dance, but there was a significant difference between religious participants (*M* = 4.19, *SD* = 1.01) and non-religious participants (*M* = 3.24, *SD* = 1.30) on how spiritual they found the Dance, *F*(1, 66) = 10.74, *p* = .002. Those with a religious orientation both anticipated and interpreted the Dance to be more spiritual than those without a religious orientation.

## Discussion

Although reports of extreme rituals anecdotally document trances and other altered states of consciousness [7, 8], this study is among the first to have empirically documented changes in proxy measures of altered states of consciousness during an extreme ritual in a naturalistic setting. Participants in a ritual involving body piercing with hooks or weights pulling against the piercings showed significant decrements in Stroop performance, which represented decreased executive functioning consistent with transient hypofrontality [16]. Moreover, this effect was not isolated to individuals receiving painful piercings but was also evidenced in individuals who were not pierced. Similarly, both pierced and non-pierced participants reported experiencing flow during the ritual. These altered states of consciousness findings add to the current body of literature and might help to further account for (a) the prevalence of extreme rituals, (b) the willingness of people to subject themselves to such rituals, and (c) the processes whereby some of the previously identified effects of extreme rituals might take place (e.g., the finding that extreme rituals increase prosocial behavior [5] could be explained by the altered states of consciousness identified by our research).

As noted above, evidence of two altered states of consciousness (transient hypofrontality and flow) appeared in both focal ritual participants (i.e., pierced dancers) and in participants adopting other roles (e.g., drummers, event leaders, observers). The use of two measures, one direct self-report and one indirect objective task, helps to provide convergent validity of the participants’ experiences of altered states of consciousness during the ritual. Interestingly, it appeared that individuals who would eventually get pierced during the Dance demonstrated marginally significant greater impaired executive functioning at baseline compared to individuals who did not get pierced during the Dance. It is possible that individuals anticipating the Dance were already experiencing some cognitive changes based on their mental preparation for the Dance. Anecdotally, some participants planning to get pierced shared with the researchers that they had engaged in meditation before arriving at ritual. As meditation is theorized to be an altered state of consciousness explained by transient hypofrontality [16] it is possible this might have contributed to these baseline differences.

There was one notable exception to the increase in Stroop latency during the Dance, and this was the piercers. Although the relatively small number of piercers in our sample precludes a confident interpretation, it is notable that piercers were the only group who did not show evidence of executive function impairment during the Dance, and they also exhibited the highest levels of flow. The technical precision needed to safely perform piercings might have required piercers to maintain attentional focus not required of other roles (e.g., the drummers)—attentional focus that manifested in the maintenance of executive function and the experience of flow. This distinction between the altered states exhibited by piercers and those exhibited by other roles may parallel the role-specific altered states discussed by providers (i.e., top space) versus recipients (i.e., subspace, bottom space) of stimulation in consensual sadomasochistic scenes [35, 39]. Similarly, other research on transient hypofrontality suggests that there are many practical implications of impaired executive functioning for individuals in various occupations (e.g., fire fighters or police) on their ability to make informed, logical decisions while they experience physical exertion and at what point physical exertion would become problematic [17]. It would be of interest to further investigate how individuals holding various roles in extreme rituals experience altered states differently and how this is related to the activities they engage in during the ritual. We want to highlight, however, that the sample sizes for each non-pierced role were very small, and we were, therefore, not able to statistically examine the effects of the Dance for each one. Nevertheless, the pattern of means for each role is suggestive and should be considered when planning future research on extreme rituals in naturalistic settings. Given the difficulty of recruiting a sufficient sample size of participants in certain roles (e.g., piercers) within one ritual for statistical analysis, aggregation across multiple rituals could be of use in overcoming this potential sample size limitation.

The results from the other psychological self-report measures are consistent with the theorized positive effects of experiencing an altered state of consciousness [6, 22] and with previously documented studies on extreme rituals [2, 10]. In particular, Dance participants reported feeling less negative affect and psychological stress from before the Dance to afterwards. Contrary to predictions, however, positive affect did not increase. One interpretation of these results might be that the Dance of Souls appeared to help to relieve negative emotions more so than to increase positive ones. Alternatively, it may have been that individuals were already experiencing a high level of positive affect from other conference activities and the ritual did not increase these feelings above and beyond baseline levels. It would be of interest for future studies to examine a similar type of hook-pull ritual independent of a weekend conference. Furthermore, baseline levels of negative affect and psychological stress could have been elevated as a result of nervous anticipation of the Dance, and the observed decline might not have been the result of the ritual itself so much as the result of the easing of this nervous anticipation. Additionally, the perspective that extreme rituals help to promote social bonds [8] is consistent with our findings that feelings of intimacy increased from before to after the event.

Contrary to our predictions and somewhat inconsistent with previous literature, effects sufficient to detect differences by ritual role were not observed with these self-reported affective measures in this sample. Indeed, the reductions in stress and negative affect and the increases in intimacy occurred regardless of whether or not participants had received painful temporary piercings. Thus, experiencing the intensity of the piercings during this extreme ritual did not appear to be necessary for participants to receive these benefits of the ritual. It may be that the collective nature of the event facilitated similar effects for all those involved. That said, it may also be that the presence of pierced individuals might have been necessary for the non-pierced individuals to have experienced the effects. The non-pierced Dance participants engaged in activities that varied in their intensity level (e.g., drummers versus observers), and it would be of interest for future studies to differentiate the levels of involvement required to receive affective benefits of the ritual. It is worth highlighting that a substantially greater sample size would be needed before making strong conclusions about a lack of group differences during this type of extreme ritual. One effect that was specific to pierced dancers was the increase in cortisol from before to during the Dance. This increase in cortisol is not surprising, given the pain of receiving and pulling against the piercings. But this significant increase in physiological stress stands in contrast to the significant decrease in psychological stress that occurred at the same time. This seeming disconnect between the physiological and the psychological parallels the findings of Xygalatas and colleagues [4] that fire-walkers reported reductions in experienced arousal even as their actual heart rates increased. We suspect that such physiological/psychological discrepancies might be an indicator of the types of altered states of consciousness that extreme rituals sometimes produce.

Results from the pre- and post-Dance surveys demonstrated that participants viewed the Dance as more spiritual than sexual or sadomasochistic, despite the event occurring at the conclusion of a leather/BDSM conference. Theorists and researchers have noted that both context and individual characteristics play a role in an individual’s interpretation of a ritual [6, 10]. This perception of the Dance as primarily spiritual might stem from a number of sources. First, given the historical nature of the Dance of Souls having been inspired by the Plains Native American Sundance (a.k.a. O-Kee-Pa ceremony) [1] and the Hindu Thaipusam festival of the Tamil communities, it may be that participants went into the experience anticipating the ritual to be spiritual and thus shaped their experience to reflect this expectation (indeed, they did anticipate that the event would be more spiritual than sexual or sadomasochistic). Second, it may be that the experience of an altered state of consciousness during an extreme ritual leads participants to interpret the event as more spiritual. Furthermore, if participants are made aware ahead of time that the experience of altered states is a common occurrence during the ritual, this too may lead participants to anticipate the event as spiritual in nature. Also, people schematic for spirituality may tend to interpret these types of experiences in a spiritual way. For example, in the Jegindo et al. study [10], it was demonstrated that participants’ religious beliefs and spiritual expectations helped them cope with the pain of the ritual and experience a positive outcome from the event. In this study, those with a religious orientation both expected and found the Dance to be more spiritual compared to those without a religious orientation. Given our data, however, we were only able to dichotomize religious orientation. It therefore would be beneficial for future work to investigate spiritual orientation using a continuous measure. Interestingly, the results indicated that sexual arousal increased from before to during the ritual, although participants did not interpret the Dance as particularly sexual. Additional research is needed to investigate whether this sexual arousal increase was a result of context, individual differences, or another variable, such as an increase in general physiological arousal. Future research that examined extreme rituals in different contexts could help to determine how the affective and physiological effects are tied to participants’ conceptualization of the event. In addition, future research could profitably examine which elements of the ritual (the pain of the piercings, the rhythm of the drumming, the spiritual framing of the event) contributed to the observed effects. As an example, Fisher and colleagues [40] conducted an investigation on how synchronous body movement during nine collective rituals in naturalistic settings affected participants’ prosociality, and the results demonstrated that rituals involving high body synchrony (deliberately matching each other’s movement and vocalizations in time for more than 30 minutes) led to greater prosocial behavior, enhanced feelings of oneness with others, and more trust among group members. In the present study, the synchrony observed between the pierced participants and the non-pierced participants (e.g., drummers) might have contributed to these results. A hook-pull outside of a collective context might lead to different results; for example, when one person individually engages in a hook-pull ritual while being observed by others.

Further, although we interpret the decrements in Stroop performance as supporting transient hypofrontality [16], they might also represent ego depletion or fatigue [19, 41] It would be useful for future studies on extreme rituals to include measures of ego-depletion (e.g., a hand-grip task) or fatigue to determine if these alternate explanations can account for the results. Future research that tests these competing explanations and other potentially relevant psychological phenomena (e.g., deindividuation, mindfulness) would be of value. Others have posited that distraction might be a potential explanation for impaired executive functioning. In the Dietrich and Sparling [19] study, exercising participants did not show decrements on general intelligence performance, and the authors argued that impaired executive functioning is not likely due to participants simply experiencing divided attention or distraction during exercise, otherwise performance on the general cognitive measures would have been impeded as well.

As with Fischer et al. [2] and Xygalatas et al. [5], the present study examined the effects of a ritual already taking place in the field. Although we were able to obtain a number of physiological and psychological measures before, during, and after the ritual, random assignment was not possible. This is a universal limitation of field studies of this type. Although such studies provide a high level of ecological validity, this ecological validity comes at a cost of internal validity. Additionally, because we could not use random assignment, it is possible that the observed effects are due to self-selection or volunteer bias. Last, at least in regards to the self-report measures, such as flow and affect, participants might have been motivated to respond in socially desirable ways. Therefore, these results should be interpreted with caution.

The social sciences are concerned, in part, with understanding extreme behavior. Despite being widely practiced, extreme rituals can be perceived from outside the cultural context in which they take place as dangerous and unhealthy. These perceptions have the potential to lead to problematic assumptions about the individuals who engage in these behaviors such as attributions of pathology and a need for intervention. Without better knowledge or awareness, these attributions could lead to stigmatization and discrimination. Empirical evidence can provide insight into the motivations for engaging in such mysterious and potentially troubling behavior. To this end, this study demonstrates that extreme rituals are associated with subjectively pleasant altered states of consciousness. These altered states may help to explain why individuals are motivated to endure these rituals and why these rituals endure through time. These types of insight have the potential to facilitate better communication and understanding between those who engage in extreme rituals and those who do not.

## Materials and Methods

The Dance of Souls took place in a large ballroom on the final day of the 2014 Southwest Leather Conference (SWLC), an annual four-day conference in Phoenix, Arizona. Approximately 180 people participated in the Dance of Souls. Of these, 83 enrolled in the study (49 women, 32 men, 2 transgender individuals; *M*_age_ = 48.02, *SD*_age_ = 10.85, *Range* 24–69). The majority of participants were Caucasian (83.1%, 7.2% mixed ethnicity, 2.4% Native American, 2.4% Hispanic, 1.2% Asian, 1.2% African American, 1.2% other, 1 missing), but there was a greater diversity in regards to sexual orientation (42.2% bisexual or heteroflexible, 22.9% heterosexual, 13.3% gay/lesbian, 12.0% queer, 8.4% pansexual, 1 missing) and religiosity (24.1% “spiritual,” 13.3% Atheist/Agnostic/None, 16.9% Pagan/Shamanic/Native American, 14.5% mixed religions, 7.2% Christian/Catholic, 4.8% Buddhist/Hindu, and 19.3% missing or not clear). The majority of participants (63.9%) also indicated that they had participated in other hook-pull/ball-dances (like the Dance of Souls or other similar events) prior to the data collection. After they were asked what role they were planning on taking on for the Dance on the pre-Dance survey, they were asked how experienced they were in that role on a 1 (*no or very little experience*) to 5 (*very experienced*), and participants reported a mean of 2.96 (*SD* = 1.57).

### Procedure and Description of Ritual Setting

The Dance of Souls took place on the last day of the Southwest Leather Conference in Phoenix, Arizona. Beginning on Friday evening, the second night of the conference, a table was set up by the researchers to invite individuals to sign up for the study. This table was located in the hallway outside of the main conference rooms, within a well-trafficked area. The researchers maintained a presence at this table throughout the duration of the conference and wore name badges indicating their institutional affiliation. If an individual was interested in participating in the study, they were given an informed consent form to read, and if they agreed to participate, they chose a unique ID which, along with a participant number, was used to track their participation throughout the study. Participants were informed they could participate in the study as much or as little as they liked; it was strongly emphasized that they were under no obligation to complete all of the measures.

After participants provided verbal informed consent (we requested and received permission to obtain informed consent verbally because of the potential risk a signed informed consent form could have posed to participants), they completed a demographic survey. Participants then completed a practice Stroop test with feedback on correct/incorrect answers before they completed a baseline Stroop test without feedback. A researcher entered the participant’s unique ID into the Stroop application and then the participant completed the test near a researcher, who was available for any problems the participant had while completing the task.

Conference attendees could enroll in the study (and complete the demographic and take the practice and baseline Stroop test) at any point until an hour prior to the beginning of the Dance. Participants were asked to return to the researchers’ table an hour before the Dance began to complete the pre-Dance survey and provide a saliva sample. This table was situated outside the ballroom where the Dance would take place.

The saliva sample was conducted by giving participants a small, sterile cylindrical sponge to put in their mouth between the gum and cheek for one minute. After a minute, the participants were then handed a clear vial by a research assistant (the vial had previously been marked with their participant number) and instructed to place the sponge into the vial using their mouth and tongue. Participants then closed the vial and a researcher placed the vial into a cooler filled with ice. This procedure was repeated during the Dance and afterwards.

After completing the pre-Dance survey, participants were asked if they would allow a researcher to approach them during the Dance to complete the during-Dance measures (a saliva sample and a Stroop test). The researchers informed the participants that they would not be interrupted while they were physically engaged with another participant (either dancing or piercing) and could waive the researcher away if they were not ready or did not want to complete the during-Dance measures at that time. The researchers explained that the interruption could take the form of eye contact, a hand wave, and/or a gentle touch on the shoulder. All participants agreed to being interrupted. Participants were then given a red wristband with their participant number on it to indicate that they had agreed to being interrupted by researchers for during-Dance data collection.

The ballroom was a large, rectangular room with piercing stations set up on one end, drummers along one long wall, tables with snacks along the opposite long wall, and a four-posted wooden structure in the middle of the room. The wooden structure had attachment points to which dancers could clip their ropes, thus providing an inanimate object to pull against. There were 10 piercing stations, and these stations consisted of a piercer, a piercing assistant, two chairs (one for the piercer and one for the piercee) and table for the necessary piercing materials. The piercers conducted the piercing, and once pierced, individuals moved to the side of the piercing stations to have, if desired, ropes tied to their hooks by a member of the piercing team. The researchers set up a table in one of the unoccupied corners of the ballroom to collect during-Dance measures.

The Dance lasted approximately 4 hours (approximately 2:00pm to 6:00pm). Slow, light drumming alerted individuals that the Dance was beginning; participants made their way into the ballroom and sat on the floor. The Dance leaders welcomed everyone, explained the logistical procedures and safety protocols, and made introductory remarks. During this time, the piercing team prepared their materials. At the conclusion of the speeches, participants were free to start dancing (the drumming got much louder) and they were invited to form a line to get pierced. Dancers could get pierced by the next available piercer or could request a certain piercer to pierce them. Once most individuals who wanted to be pierced were pierced, some members of the piercing team asked other piercers to pierce them and then they began to dance as well.

Participants with filaments and weights tended to dance by moving their bodies rhythmically, which caused the weights to pull against the piercings. Participants with hooks and ropes tended to dance by connecting the ropes to other participants (pierced and non-pierced) and pulling against them or by connecting the ropes to the central structure and pulling against it.

At 3:07pm, researchers began collecting during-Dance measures from individuals on the piercing team (Stroop and saliva sample). We ensured that we waited at least 20 minutes to collect during-Dance saliva samples from participants after they had started actively engaging in their activity (e.g., after piercers started piercing, after pierced participants had gotten pierced) because there is a latency of approximately 20 miutes between a stimulus and the corresponding change in salivary cortisol [42]. The researchers asked piercers and piercing assistants to complete the during-Dance measures when they were not actively piercing someone. The researchers would attempt to make eye contact with the piercing team member, and if the researchers received a nod they approached the participant with a saliva collection vial and a tablet with the Stroop test. When the piercer or piercing assistant had completed the measures the researchers removed their wristband.

Participants in other roles completed during-Dance measures at the researchers’ table starting at 3:45pm. Most of these participants were interrupted during the Dance by a researcher and brought to the researchers’ table to complete the during-Dance measures. Some participants approached the table on their own accord and completed the during-Dance measures at that time. When participants had completed the measures the researchers removed their wristband.

At any point during the Dance participants could ask a piercer to remove their hooks or filaments. The Dance officially concluded when the drumming stopped and Dance leaders made closing remarks. If pierced participants had not already had their piercings removed they were instructed to do so at that time. Immediately following the Dance, starting at approximately 6pm, researchers attended to the tables outside the ballroom with the post-Dance survey and the post-Dance saliva sample. The majority of participants completed the post-Dance measures directly after the Dance and upon exiting the ballroom.

Not all participants completed the materials in full during data collection, however, and many participants were missing at least some data. In an effort to rectify these omissions, three months after the conference was over the conference organizers sent an email to all the Dance attendees with a link to an online version of the surveys. The goals were to (a) collect additional information from existing participants (role clarification and a measure of spirituality), (b) allow existing participants (identified by original codename) to complete any missing surveys (pre or post), and (c) allow new participants to fill out the pre and post-Dance surveys. If existing participants filled out a duplicate version of the pre or post-Dance survey, their responses were averaged in the dataset. It was not possible to collect Stroop and saliva samples in this manner.

#### Measures

The pre-Dance survey first asked individuals to identify the role(s) they intended to play during the Dance and “how spiritual”, “how sadomasochistic” and “how sexual” they anticipated the Dance would be for them (1 = *not at all*, 5 = *extremely*). Then they were asked to describe their relationship with the other people participating in the Dance using the Inclusion of Other in Self (IOS) scale [15], a measure of intimacy. Next, participants responded to the Positive and Negative Affect Schedule (PANAS) [14], rating how much they currently felt 10 positive emotions (e.g., interested, excited, strong) and 10 negative emotions (e.g, distressed, upset, guilty) (1 = *very slightly or not at all*, 2 = *a little*, 3 = *moderately*, 4 = *quite a bit*, and 5 = *extremely*). Two additional items were included: “sexually aroused” and “stressed.”

The Stroop tests were administered on tablet devices (i.e., iPads, Android tablets) and consisted of 40 trials in which a character string (*red*, *blue*, *green*, *yellow*, or *xxxx*) was displayed in red, blue, green, or yellow font color (each of the 20 combinations was presented twice). Participants attempted to ignore the semantic meaning of the word and to respond to each by pressing a button on the screen indicating the font color (labeled in black type *red*, *blue*, *green*, and *yellow*). Stroop scores reflect the difference between the mean latencies on incongruent trials (e.g., *red* in green type) and the mean latencies on congruent trials (e.g., *red* in red type, *xxxx* in any color of type). Trials with erroneous responses and trials with latencies > 3 SD from a test’s mean latency were removed [43]. Alternative analyses that include these erroneous and >3 SD trials do not substantively change the results. Participants who completed both Stroop tests (baseline and during the event) were included in the Stroop analyses. A bug in the Stroop software caused duplicate trials to be written to the output file for 33/118 Stroop tests. These trials were identified and removed prior to data analysis.

Saliva sample assays were performed using immunoassay kits purchased from Salimetrics, LLC. All saliva samples were stored on wet ice during the collection procedures and then at -65°C for later analysis. Immediately prior to assaying, samples were thawed and centrifuged (1,500 × *g* at 3,000 rpm for 15 min) to separate saliva from any other matter. Each sample was analyzed for cortisol, without freezing the sample again. Each sample, standard or assay diluent, was pipetted into plates pre-coated with antibodies for cortisol. The standard for cortisol ranged from 0.007 μg/dl (0.20 nmol/l) to 1.8 μg/dl (49.7 nmol/l), with an average 3.60% intra-assay and 2.57% inter-assay coefficient of variation. All samples were assayed in duplicate. Sample duplicates that exceeded the standard curve were diluted with sample buffer and re-assayed the same day. Likewise, sample duplicates that varied more than 20% were re-assayed on another plate the same day and the new values reported [44]. The immunoassays were conducted in accordance with the directions from the Salimetrics, LLC hormone kits. Briefly, the conjugate was added to each well with the sample, standard or assay diluent. After 60 min, the plate was rinsed and 200 ll of tetramethylbenzidine (TMB) solution was added. The reaction was stopped after 30 min with sulfuric acid and the plate read with a BioRad E1A Reader Model 2550 within 10 min at wavelength 450 nm. After the saliva samples were analyzed it was discovered that two participants had extreme cortisol levels that were above the normal curve, and these were removed. Salimetrics also recommended removing samples with cortisol levels greater than three standard deviations above the mean. This led to the removal of seven additional samples, leaving 178 samples in the final dataset.

The post-Dance survey included the Flow State Scale (FSS) [30] (36 items, 1 = *strongly disagree*, 2 = *disagree*, 3 = *neither agree nor disagree*, 4 = *agree*, 5 = *strongly agree)*. The FSS measures nine dimensions of flow: Challenge-Skill Balance (a feeling of balance between the demands of the situation and personal skills), Action-Awareness Merging (a feeling of being so involved that one’s actions are automatic), Clear Goals (a feeling of knowing exactly what is needed in the situation on a moment to moment basis), Unambiguous Feedback (immediate and clear feedback that is seamlessly integrated into the ongoing activity), Concentration on Task at Hand (a feeling of being totally focused), Sense of Control (an empowering feeling of being in control and being free of the fear of failure), Loss of Self-Consciousness (concern for the self disappears), Transformation of Time (a change in how the passage of time is perceived, either faster, slower, or a lack of awareness), and Autotelic Experience (a feeling of intrinsic reward, task is enjoyable in and of itself). Participants were instructed to respond to each item in terms of how they felt during the event. We included an “N/A” option for participants who felt an item was not relevant to their experience. 2.7% of the total responses were N/As. For analysis, N/A responses were re-coded as missing data.

The post-Dance survey also included the PANAS, self-reported stress, self-reported sexual arousal, the IOS, and how spiritual, sadomasochistic, and sexual the Dance was.

## References

[1] Catlin G.O-Kee-Pa, a religious ceremony; and other customs of the Mandans. Philadelphia: J. B. Lippincott and Co; 1867.

[2] Fischer R, Xygalatas D, Mitkidis P, Reddish P, Tok P, Konvalinka I, et al The fire-walker’s high: Affect and physiological responses in an extreme collective ritual. PloS ONE. 2014; 9: e88355 10.1371/journal.pone.0088355 24586315 PMC3930548

[3] Konvalinka I, Xygalatas D, Bulbulia J, Schjoedt U, Jegindø EM, Wallot S, et al Synchronized arousal between performers and related spectators in a fire-walking ritual. Proc Natl Acad Sci. 2011; 108: 8514–8519. 10.1073/pnas.1016955108 21536887 PMC3100954

[4] Xygalatas D, Schjoedt U, Bulbulia J, Konvalinka I, Jegindø EM.Autobiographical memory in a fire-walking ritual. J Cogn Cult. 2013; 13: 1–16.

[5] Xygalatas D, Mitkidis P, Fischer R, Reddish P, Skewes J, Geertz AW, et al (2013) Extreme rituals promote prosociality. Psychol Sci 24: 1602–1605. 10.1177/0956797612472910 23740550

[6] Ward C.Thaipusam in Malaysia: A psycho-anthropological analysis of ritual trance, ceremonial possession and self-mortification practices. Ethos. 1984; 12: 307–334.

[7] Simons RC,producer and director, Pfaff G,film maker. Floating in the air, followed by the wind [Film]; 1973. United States: Michigan State University and University of Malaya.

[8] Xygalatas D. Trial by fire: From fire-walking to the ice-bucket challenge, ritual pain and suffering forge intense social bonds. Aeon. 2014. Available: http://aeon.co/magazine/society/how-extreme-rituals-bond-us-for-life/. Accessed 23 October 2014.

[9] Tart C.States of consciousness. New York: Dutton; 1975.

[10] Jegindø EME, Vase L, Jegindø J, Geertz A.Pain and sacrifice: Experience and modulation of pain in a religious piercing ritual. Int J Psychol Relig. 2013; 23: 171–187.

[11] Deeley Q, Oakley DA, Walsh E, Bell V, Mehta MA, Halligan PW. Modeling psychiatric and cultural possession phenomena with suggestion and fMRI. Cortex. 2014; 53: 107–119. 10.1016/j.cortex.2014.01.004 24632378

[12] Schjoedt U, Stødkilde-Jørgensen H, Geertz AW, Roepstorff A.Highly religious participants recruit areas of social cognition in personal prayer. Soc Cogn Affect Neurosci. 2009; 4: 199–207. 10.1093/scan/nsn050 19246473 PMC2686228

[13] Musafar F. Ecstatic rites: Ball dance. Body Play & Modern Primitives Quarterly, 1(4). 1992; Menlo Park, California: Insight Books, pp. 18–25.

[14] Watson D, Clark LA, Tellegen A. Development and validation of brief measures of positive and negative affect: the PANAS scales. J Pers Soc Psychol. 1988; 54: 1063 3397865 10.1037//0022-3514.54.6.1063

[15] Aron A, Aron EN, Smollan D. Inclusion of Other in the Self Scale and the structure of interpersonal closeness. J Pers Soc Psychol. 1992; 63: 596.

[16] Dietrich A.Functional neuroanatomy of altered states of consciousness: The transient hypofrontality hypothesis. Conscious Cogn. 2003; 12: 231–256. 12763007 10.1016/s1053-8100(02)00046-6

[17] Del Giorno JM, Hall EE, O’Leary KC, Bixby WR, Miller PC.Cognitive function during acute exercise: a test of the transient hypofrontality theory. J Sport Exerc Psychol. 2010; 32: 312–323. 20587820 10.1123/jsep.32.3.312

[18] Dietrich A.Transient hypofrontality as a mechanism for the psychological effects of exercise. Psychiatry Research. 2006; 145: 79–83. 17081621 10.1016/j.psychres.2005.07.033

[19] Dietrich A, Sparling PB.Endurance exercise selectively impairs prefrontal-dependent cognition. Brain Cogn. 2004; 55: 516–524. 15223198 10.1016/j.bandc.2004.03.002

[20] Sheehan PW, Donovan P, MacLeod CM.Strategy manipulation and the Stroop effect in hypnosis. J Abnorm Psychol. 1988; 97: 455–460. 3204232 10.1037//0021-843x.97.4.455

[21] Hogervorst E, Riedel W, Jeukendrup A, Jolles J. Cognitive performance after strenuous physical exercise. Percept Mot Skills. 1996; 83: 479–488. 8902021 10.2466/pms.1996.83.2.479

[22] Sibley BA, Etnier J L, Le Masurier GC. Effects of an acute bout of exercise on cognitive aspects of Stroop performance. J Sport Exerc Psychol. 2006; 28: 285–299.

[23] Csikszentmihalyi M.Flow: The psychology of optimal experience. New York: Harper Collins; 1990.

[24] Csikszentmihalyi M, Csikszentmihalyi I.Optimal experience: Psychological studies of flow in consciousness. Cambridge: Cambridge University Press; 1988.

[25] de Manzano Ö, Theorell T, Harmat L, Ullén F. The psychophysiology of flow during piano playing. Emotion. 2010; 10: 301 10.1037/a0018432 20515220

[26] Jackson SA. Factors influencing the occurrence of flow state in elite athletes. J Appl Sport Psychol. 1995; 7: 138–166.

[27] Jackson SA. Toward a conceptual understanding of the flow experience in elite athletes. Res Q Exerc Sport. 1996; 67: 76–90. 8735997 10.1080/02701367.1996.10607928

[28] Rathunde K, Csikszetnmihalyi M. Middle school students' motivation and quality of experience: A comparison of Montessori and traditional school environments. AM J Educ (Chic III). 2005; 111: 341–371.

[29] Jackson SA, Marsh HW. Development and validation of a scale to measure optimal experience: The flow state scale. J Sport Exerc Psychol. 1996; 18: 17–35.

[30] Jackson S, Eklund B, Martin A. The FLOW manual: The manual for the Flow Scales. Queensland, Australia: Mind Garden, Inc; 2010.

[31] Fischer R, Xygalatas D.Extreme rituals as social technologies. J Cogn Cult. 2014;14: 345–355.

[32] Bulbulia JA, Xygalatas D, Schjoedt U, Fondevila S, Sibley CG, Kovalinka I. Images from a jointly-arousing collective ritual reveal affective polarization. Front Psychol. 2013; 4: 1–11. 24399979 10.3389/fpsyg.2013.00960 PMC3872332

[33] Whitehouse H, Lanman JA.The ties that bind us. Curr Anthropol. 2014; 55: 674–695.

[34] Kirschbaum C, Hellhammer DH.Salivary cortisol in psychoneuroendocrine research: Recent developments and applications. Psychoneuroendocrinology. 1994; 19: 313–333. 8047637 10.1016/0306-4530(94)90013-2

[35] Wiseman J. SM 101: A realistic introduction. Gardena, CA: Greenery Press; 1998.

[36] Sagarin BJ, Cutler B, Cutler N, Lawler-Sagarin KA, Matuszewich L. Hormonal changes and couple bonding in consensual sadomasochistic activity. Arch Sex Behav. 2009; 38: 186–200. 10.1007/s10508-008-9374-5 18563549

[37] Baldwin G.Ties that bind: The SM/leather/fetish erotic style. San Francisco: Daedalus Publishing Company; 1993.

[38] Easton D, Hardy JW.The new bottoming book. Emeryville, CA: Greenery Press; 2001.

[39] BDSM FAQ Glossary (n.d.). Available: http://www.reddit.com/r/bdsmfaq/comments/wz9wv/glossary_part_ii/. Accessed 9 April 2014.

[40] Fischer R, Callander R, Reddish P, Bulbulia J.How do rituals affect cooperation? Hum Nat. 2013; 24: 115–125. 10.1007/s12110-013-9167-y 23666518

[41] Baumeister RF, Bratslavsky E, Muraven M, Tice DM.Ego depletion: Is the active self a limited resource? J Pers Soc Psychol. 1998; 74: 1252–1265. 9599441 10.1037//0022-3514.74.5.1252

[42] Cook NJ, Read GF, Walker RF, Harris B, Riad-Fahmy D.Salivary cortisol and testosterone as markers of stress in normal subjects in abnormal situations In Kirschbaum C, Read GF, Hellhammer DH, editors.Assessment of hormones and drugs in saliva in biobehavioral research. Seattle: Hogrefe & Huber; 1992, pp. 147–162.

[43] Linnman C, Carlbring P, Ahman A, Andersson H, Andersson G. The Stroop effect on the internet. Comput Human Behav. 2006; 22: 448–455.

[44] Kivlighan KT, Granger DA, Schwartz EB, Nelson V, Curran M, Shirtcliff EA. Quantifying blood leakage into the oral mucosa and its effects on the measurement of cortisol, dehydroepiandrosterone, and testosterone in saliva. Horm Behav. 2004; 46: 39–46. 15215040 10.1016/j.yhbeh.2004.01.006

